# Sublethal effects of buprofezin on development and reproduction in the white-backed planthopper, *Sogatella furcifera* (Hemiptera: Delphacidae)

**DOI:** 10.1038/s41598-017-17190-8

**Published:** 2017-12-05

**Authors:** Ehsan Ali, Xun Liao, Peng Yang, Kaikai Mao, Xiaolei Zhang, Muhammad Shakeel, Abdalla M. A. Salim, Hu Wan, Jianhong Li

**Affiliations:** Hubei Insect Resources Utilization and Sustainable Pest Management Key Laboratory, College of Plant Science & Technology, Huazhong Agricultural University, Wuhan, 430070 Hubei China

## Abstract

In the present study, the effects of sublethal concentrations of buprofezin on life-table traits of *S. furcifera* were evaluated for two consecutive generations (F0 and F1). Our results exhibited that the fecundity, life span (longevity) and hatchability of the F0 and F1 generations were significantly decreased at LC_30_ compared to the control. However, copulation was not significantly affected for the F0 or F1 generations at sublethal concentrations. The female life span was affected negatively at both treatments in F0 and at LC_30_ in F1, compared to the control. Furthermore, significant effects of the sublethal concentrations were found on the developmental rate of all instars except the 3^rd^ instar of F1. However, the pre-adult period, total pre-oviposition period (TPOP) and adult pre-oviposition period (APOP) significantly increased in F1 individuals at LC_30_ and LC_10_ compared to the control. Our findings revealed that demographic characters (survival rate, intrinsic rate of increase (*ri*), finite rate of increase (λ), net reproductive rate (*R*_0_), and gross reproductive rate (*GRR*)) of the F1 generation (from F0 parents) significantly decreased compared to the untreated group; however, the generation time (*T*) increased at LC_10_. Therefore, the results suggested that buprofezin could adversely affect individuals in the successive generation.

## Introduction

Rice (*Oryza sativa* L.) is the 2^nd^ main food source for more than half of the world’s population and affects the livelihood and income of one hundred million people^1^. The white- backed planthopper (WBPH), *Sogatella furcifera* (Horvath), is a destructive rice pest throughout Asia, and it causes serious yield losses by sucking cell sap and ovipositing in rice stems^2^. Recently, outbreaks of the WBPH have damaged rice crops at the immature growth stage by transmitting southern rice black-streaked dwarf virus (SRBSDV). This virus was first reported at a location in Yangxi, Guandong Province, China in 2001^3^. *S. furcifera* as well as the viral disease, causes heavy yield losses of rice in China and elsewhere in Asia^3,4^.

Buprofezin, is chitin synthesis inhibitor developed by Nihon-Nohyaku, with very low risks to the environment and human beings, is a thiadiazine insecticide that is especially used against sucking pests, such as the planthoppers. Its worldwide uses are in China, Japan, India and Southeast Asia and its normal application is 75–100 g a.i./ha^5,6^. Its initial effect is to inhibit with chitin deposition during moulting and to cause nymphal death during cuticle shedding^5^. In addition, reduced fecundity and egg hatching have been observed after adult females were treated^5–7^. Although buprofezin lacks an acute insecticidal effect, it offers the advantage of longer residual activity against *N. lugens* nymphs than conventional insecticides^5^. Therefore, buprofezin was thought to be a unique insecticide for controlling the planthopper^5,8,9^.

Sublethal effects are defined as physiological and or behavioural effects on individuals that survived from exposure to a pesticide at sublethal concentration^10^. The insect pests are exposed to sublethal concentrations of insecticides^10^, is a common approach in agro-ecosystems due to the fact that the pesticides degraded after initial applications in field^11^. Such exposure of insecticides may also impair various key biological traits of the exposed insects through sublethal effects^10^. For example, neurophysiology processes and biochemistry, longevity, fecundity, developmental time, the sex ratio, and immune capacity^12–15^, as well as behavioural changes (like feeding, learning ability, searching, mental capacity and oviposition^10,16^. Determining the sublethal effects on arthropods is very essential for impact analyse of pesticides^17–22^.

Some scientists have suggested that the sublethal concentrations of pesticides might persuade insect outbreaks in field^23,24^. For instance, organophosphorus, pyrethroid, and organochlorine pesticides have been revealed to cause the resurgence of pests when insecticide contaminants degraded to near a low lethal level^23,24^. Some reports have found that sublethal concentrations of insecticides affect growth and increased the productivity and developmental duration in insect, for *S. furcifera*, the population growth was inhibited by sublethal concentration of triazophos, chlorantraniliprole and imidacloprid^25,26^. In various investigations, increased fecundity and survival time were also observed in *M. persicae* after treatment with sublethal concentrations of azadirachtin, imidacloprid^27^.

The use of two-sex life tables is one of the most important tools for investigating sublethal effects, particularly in life cycle studies, as it can highlight population effects that may be underestimated at the individual level^28–30^. The possible sublethal effects of buprofezin on *S. furcifera* have not yet been reported. In our study, for data interpretation two-sex life table was used to observe the sublethal effects of buprofezin, with a particular focus on the trans-generational effects on *S. furcifera*.

## Results

### Buprofezin toxicity against *S. furcifera*

The toxicity level of buprofezin to 3^rd^ instar *S. furcifera* is presented in Table 1; the estimated the LC_10_, LC_30_, LC_50_, and LC_100_ values are 0.173 mg a.i. L^−1^ (95% CI from 0.0132 to 0.483 mg a.i. L^−1^), 0.847 mg a.i. L^−1^ (95% CI from 0.217 to 1.526 mg a.i. L^−1^), 2.541 mg a.i. L^−1^ (95% CI from 0.731 to 6.647 mg a.i. L^−1^), and 332.460 mg a.i. L^−1^ (95% CI from 81.71 to 12072.15 mg a.i. L^−1^), respectively. Finally, these concentrations LC_10_ and LC_30_ were used as the sublethal concentrations for further experiments. In order to evaluate the sublethal effects, 3^rd^ instar *S. furcifera* nymph were exposed to these sublethal concentrations of buprofezin, the 120-h mortality of nymphs were 6.616 ± 0.925, 12.21 ± 0.845 and 30.11 ± 1.352% for control, LC_10_ and LC_30_ of buprofezin, respectively.

**Table 1:** LC_10_, LC_30_, LC_50_, and LC_100_ values, with corresponding 95% confidence intervals for buprofezin toxicity against *S. furcifera*.

Treatment	n^a^	LC_10_ mg a.i. L^−1^ (95% CI)^b^	LC_30_ mg a.i. L^−1^ (95% CI)	LC_50_ mg a.i. L^−1^ (95% CI)	LC_100_ mg a.i. L^−1^ (95% CI)	Slope ± SE^c^	X^2^ (df)^d^
Buprofezin	255	0.173 (0.013–0.483)	0.847 (0.217–1.526)	2.541 (0.731–6.647)	332.460 (81.710 to 12072.15)	1.099 ± 0.249	5.012(3)

^a^Number of 3^rd^ instar.

^b^95% CI, Confidence interval.

^c^SE = standard error.

^d^Chi-square testing linearity of dose-mortality responses.

### Sublethal effects of buprofezin on parental (F0) *S. furcifera*

**Figure 1:**
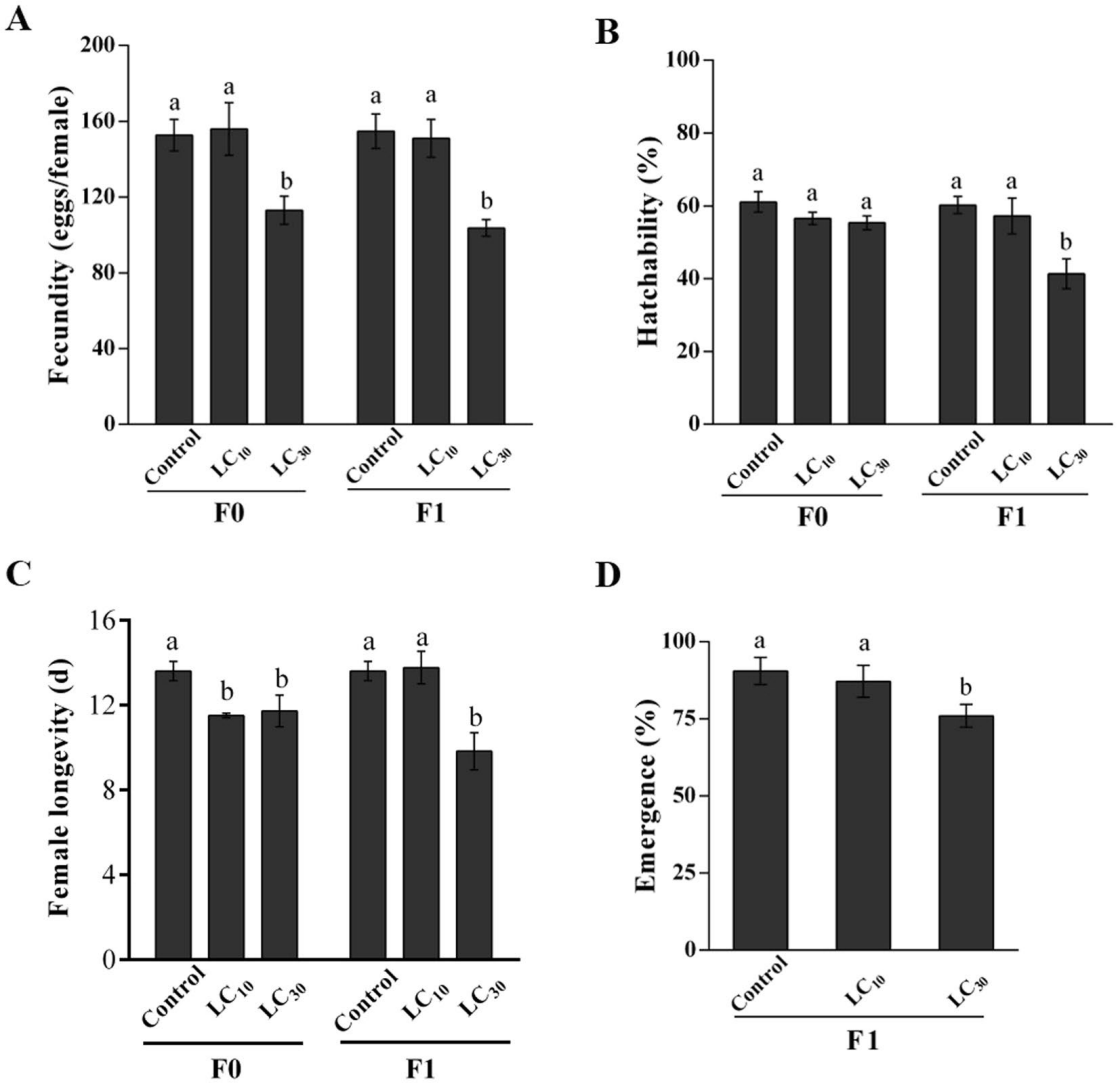
Fecundity (eggs/female) (**A**), hatchability (%) (**B**), longevity of female (days) (**C**), and emergence of adults from F1 individuals (**D**). Different letters on the bars of the histogram show significant differences.

Third instars *S. furcifera* of the F0 generation were treated with two sublethal concentrations (LC_10_ and LC_30_) of buprofezin. Fecundity and longevity of female were significantly decreased by buprofezin (Fig. 1A,C), while hatchability (Fig. 1B) and copulation (data not shown) were not affected.

### Trans-generational effects of buprofezin on individuals (F1) of *S. furcifera*

The developmental time (Table 2), fecundity, hatchability, emergence and longevity of *S. furcifera* F1 offspring produced by F0 parents treated with LC_10_ and LC_30_ buprofezin are shown in Fig. 1A–D. We found significant differences in fecundity, hatchability, longevity of female and emergence, for LC_30_ compared to LC_10_ and the control, whereas no significant effects on copulation were reported in the F1 offspring. Additionally, the developmental period of all instars (1^st^ to 5^th^ instar) of F1 individuals was significantly affected positively or negatively except for the 3^rd^ instar at LC_30_ and LC_10_ compared to the control (Table 2). The overall nymph developmental period significantly affected in the treatments compared to the control. However, the pre-adult period, APOP (adult pre-oviposition period) and TPOP (total pre-oviposition period) in the offspring of the F0 parents was significantly increased compared to the control. The total male and female longevity in F1 offspring significantly decreased at LC_30_.

**Table 2:** Developmental times of different stages for F1 individuals of *S. furcifera* when parents (F0) were treated with LC_10_ and LC_30_ of buprofezin.

Stages	Control	LC_10_	LC_30_
	Mean ± SE	Mean ± SE	Mean ± SE
1^st^ Instar	2.397 ± 0.0048b	2.900 ± 0.025a	2.214 ± 0.0036c
2^nd^ Instar	2.029 ± 0.016b	2.160 ± 0.030a	2.041 ± 0.018b
3^rd^ Instar	2.049 ± 0.021a	2.022 ± 0.012a	2.025 ± 0.014a
4^th^ Instar	2.494 ± 0.051a	2.178 ± 0.037b	2.135 ± 0.031b
5^th^ Instar	3.063 ± 0.029b	3.379 ± 0.048a	2.648 ± 0.073c
Pre-adult	16.957 ± 0.072c	18.586 ± 0.091a	18.028 ± 0.127b
Adult longevity	13.660 ± 0.504a	13.447 ± 0.409a	9.631 ± 0.399b
Longevity	28.695 ± 0.720a	29.180 ± 0.630a	24.231 ± 0.630b
T. longevity (M)	30.425 ± 0.716a	31.281 ± 0.543a	27.231 ± 0.618b
T. longevity (F)	30.810 ± 0.729b	32.839 ± 0.607a	28.077 ± 0.447c
Fecundity	147.212 ± 12.646a	165.51 ± 26.78a	93.435 ± 9.145b
APOP	1.066 ± 0.037b	2.075 ± 0.103a	2.226 ± 0.120a
TPOP	18.044 ± 0.094c	21.112 ± 0.158a	20.318 ± 0.180b

T. longevity: total longevity, T. longevity (M): total longevity of male, T. longevity (F): total longevity of female, APOP: adult pre-oviposition period, TPOP: total pre-oviposition period, Standard error of the mean (SEM) was calculated using 100,000 bootstraps resampling. A paired bootstrap test was used to detect differences between treatments and control and means followed by different letters within a row show significant difference between the control and different treatments group (at the *P* < 0.05 level).

The trans-generational effects of the sublethal concentrations (LC_10_ and LC_30_) of buprofezin on population dynamics (Table 3) were calculated with bootstrap procedure based on a life cycle. The finite rate of increase (*λ*), intrinsic rate of increase (*r*_*i*_), net reproductive rate (*R*_0_) and gross reproduction rate (*GRR*) of F1 individuals significantly decreased in the LC_30_ treatment, while the net reproductive rate and gross reproduction rate were not affected by the LC_10_. In contrast to LC_30_, LC_10_ caused a significant increase in the mean generation time (*T*) of the exposed offspring.

**Table 3:** Trans-generational effects of buprofezin on F1 population parameters of *S. furcifera*.

Parameters	Control	LC_10_	LC_30_
	Mean ± SE	Mean ± SE	Mean ± SE
*r*_*i*_	0.1722 ± 0.0054a	0.151 ± 0.0047b	0.147 ± 0.0062b
*Λ*	1.1870 ± 0.0064a	1.163 ± 0.0055b	1.158 ± 0.0072b
*R*_0_	65.855 ± 9.087a	65.768 ± 12.565a	34.699 ± 5.089b
*T*	24.255 ± 0.317b	27.597 ± 0.662a	24.009 ± 0.118b
*GRR*	144.711 ± 23.219a	173.296 ± 54.411a	58.527 ± 7.611b

*ri*: intrinsic rate of increase (d^−1^), *λ*: finite rate of increase (d^−1^), *R*_0_: net reproductive rate (offspring/individual), *T*: mean generation time (d), *GRR*: gross reproductive rate. Standard error of the mean (SEM) was calculated using 100,000 bootstraps resampling. A paired bootstrap test was used to detect differences between treatments and control and means followed by different letters within a row show significant difference between the control and different treatments group (at the *P* < 0.05 level) after using pair bootstrap test.

**Figure 2:**
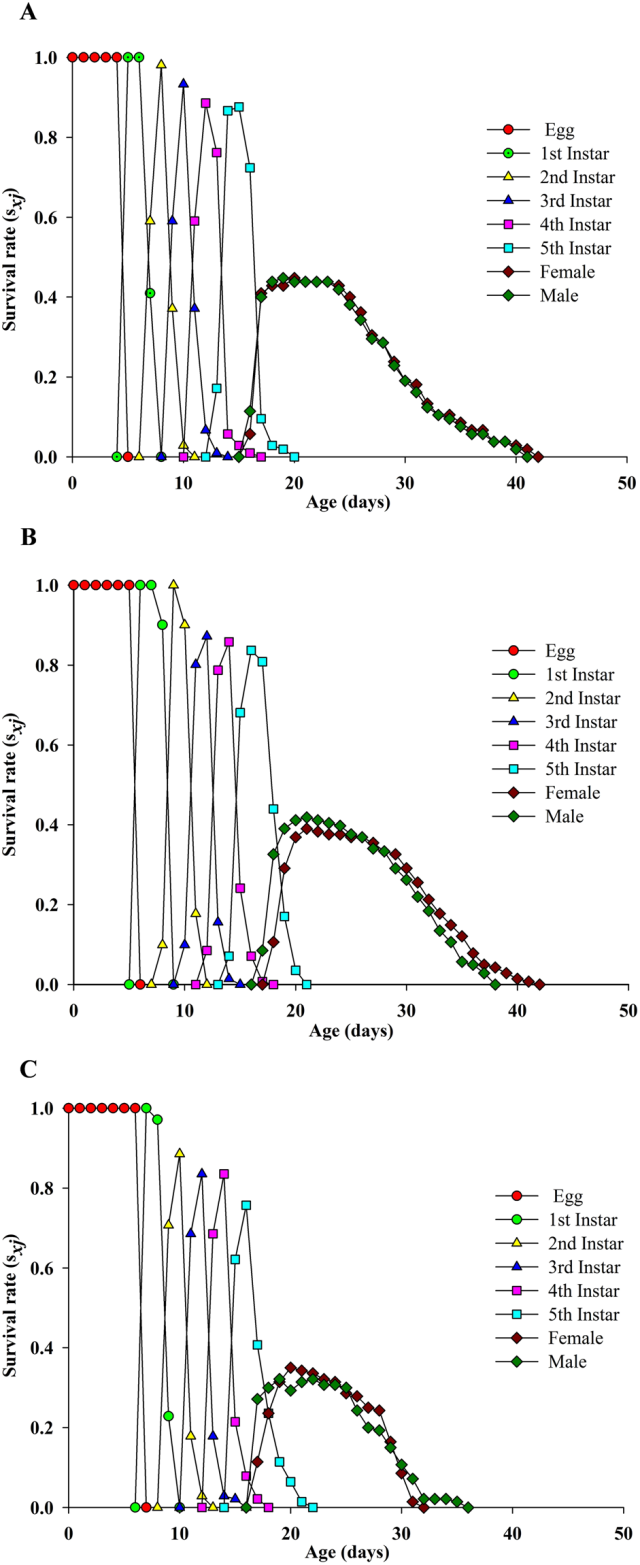
Age-stage specific survival rate (*s*_*xj*_) of *S. furcifera* individuals of the F1 generation under control conditions (**A**), treated with LC_10_ (**B**) and treated with LC_30_ (**C**) (from F0 parents).

**Figure 3:**
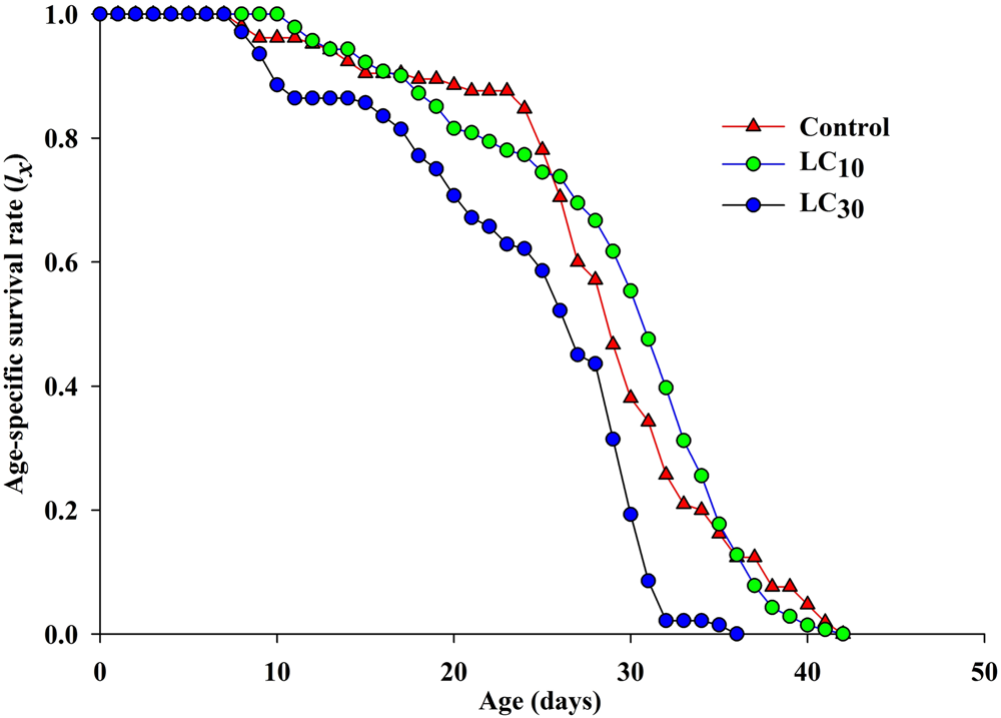
Age-specific survival rate (*l*_*x*_) of control and *S. furcifera* individuals of the F1 generation (from F0 parent exposed to the LC_10_ and LC_30_ of buprofezin).

The age-stage survival rate (*s*_*xj*_) indicated the probability that a newly laid egg will survive to age *x* and stage *j* (Fig. 2). It represents variation in the developmental rate occurring among individuals, and the coinciding projected curves clearly showed the overlapping between the different stages for buprofezin (LC_30_) and control. The plotted peaks for each developmental stage under LC_10_ and control showed almost the same pattern, with the exceptions that the curves for male adults ended earlier than those for females, while the peaks of the plotted curves at LC_30_ were not high as for the control. The peaks also showed that male and female exhibited the same survival rate for LC_10_ and the control, while they exhibited a shortened survival period at LC_30_. The curves for the female and male adults showed that sexes emerged after 16 days in the buprofezin treatment but after 15 days in the control. Likewise, the age-specific survival rate (*l*_*x*_) for the control and treatments is plotted in Fig. 3. It indicates that a similar *l*_*x*_ pattern was observed for LC_10_ and the control, while the age-specific survival rate (*l*_*x*_) declined more in the LC_30_-treated populations’ offspring at day 8 compared to the control. These results showed that the probability of new-born nymphs surviving to the adult stage was 0.80 at LC_30_ and 0.83 at LC_10,_ whereas it was 0.87 in the control.

**Figure 4:**
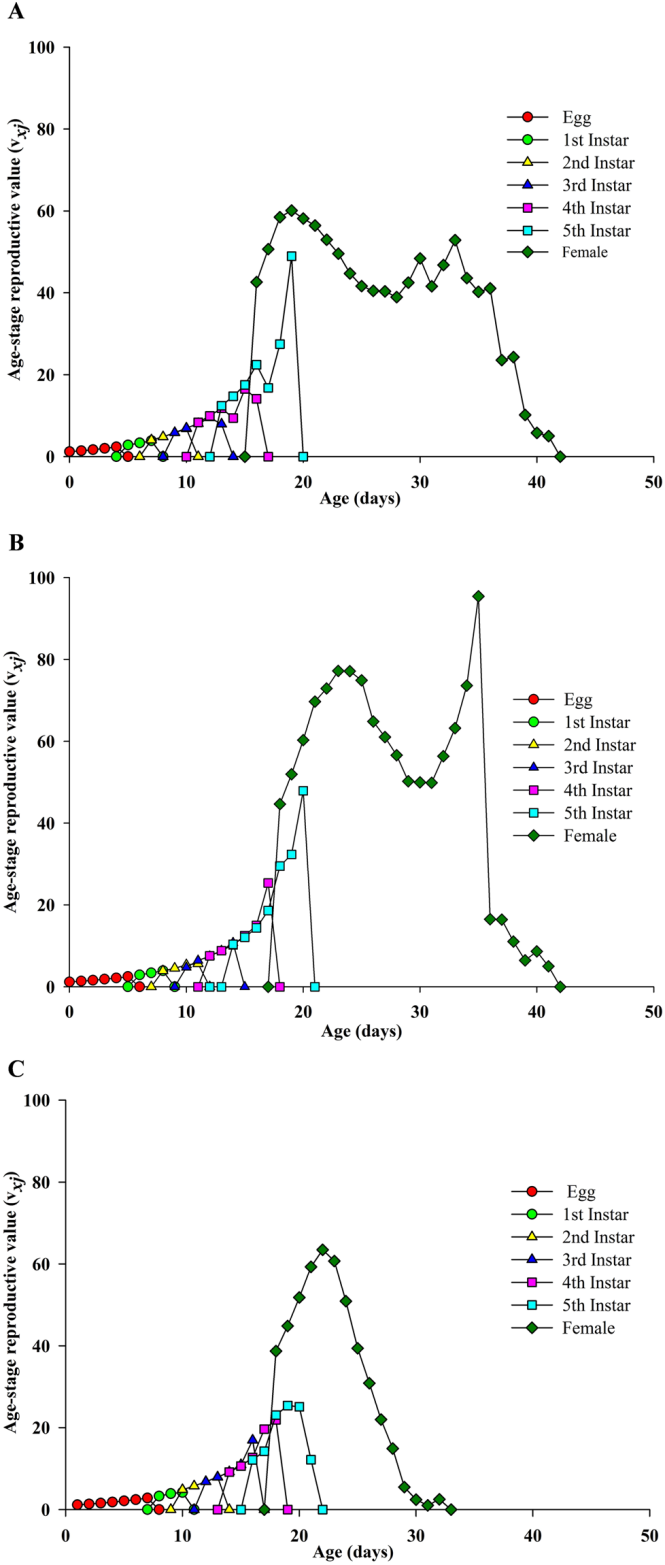
Age-stage reproductive value (*v*_*xj*_) of *S. furcifera* individuals of the F1 generation under control conditions (**A**) treated with LC_10_ (**B**) and treated with LC_30_ (**C**) (from F0 parent).

The age-stage reproductive values (*v*_*xj*_) of the buprofezin treatments showed (Fig. 4) that the *v*_*xj*_ of the LC_30_ buprofezin treatment was lower compared to LC_10_ and the control individuals in the 5^th^ instar stage. At this stage (5^th^ instar), the peak is sharper in cases of LC_10_ and the control than for LC_30_. However, in case of emerging females, the plotted curve for LC_30_ rose slightly higher and declined more rapidly compared to the control and LC_10_ but increased in an additional curve at later ages in LC_10_ treated individuals; the maximum *v*_*xj*_ value 78 d^−1^ on 23^rd^ day at LC_10_, whereas it was 60 d^−1^ on the 22^nd^ day in the LC_30_ group and 60 d^−1^ on the 19^th^ day in the control.

## Discussion

This is the first study to evaluate the effects of sublethal exposure to buprofezin on the life cycle of *S. furcifera*. Buprofezin is an insect growth regulator and is highly effective against several sucking pests^31,32^.

Insecticides are usually distributed unequally as well as subjected to degradation after application in the field so the probability of targeted and non-targeted pests to low concentration of insecticides happens very often^11,33,34^, However, studies on the effects of sublethal concentrations of insecticides on target pests are of great importance to increase their rational use^35–38^. Therefore, buprofezin may also cause a wide range of sublethal effects to pests such as in *S. furcifera*. A thorough investigation of these possible effects would help to improve IPM in rice crops.

Lethal and sublethal effects of buprofezin have been studied in some arthropods, e.g *Encarsia inaron* (Hymenoptera: Aphelinidae)^31^, *Eretmocerus mundus* Mercet (Hymenoptera: Aphelinidae)^32^, *Bemisia tabaci* (Hemiptera: Aleyrodidae)^32^. Sublethal effects such as decreased fecundity, hatchability, longevity, and copulation could result in stimulatory effects on pest population growth^10^. In the present study, we investigated the sublethal effects of buprofezin for two consecutive generations and found a significant decrease in fecundity, hatchability, emergence and longevity of *S. furcifera* females in the F1 generation at LC_30_ but not copulation, whereas in the F0 generation, a significant difference was found in fecundity and female longevity at LC_30_. Our results are in line to Zhou *et al*.^25^ reported that sublethal concentrations of imidacloprid showed significant effects on the fecundity of *S. furcifera*.

Moreover, trans-generational effects on the F1 individuals of *S. furcifera* were also found. We found that the exposure to the LC_10_ and LC_30_ of buprofezin in the parent (F0) population significantly affected the F1 offspring growth rate, especially by increasing the duration of the pre-adult stage, TPOP and APOP and decreased the longevity, total longevity of males and females and fecundity at LC_30_ and vice versa at LC_10_. These effects are related to reductions in the intrinsic rate of increase (*ri*), finite rate of increase (λ), net reproductive rate (*R*_0_), gross reproduction rate (*GRR*) and survival rate. Such effects on offspring growth have also been reported in the cotton aphid, *Aphis gossypii* Glover (Hemiptera: Aphididae) and the small brown planthopper, *Laodelphax striatellus* (Homoptera: Delphacidae) through treatment with sublethal concentrations of sulfoxaflor and thiamethoxam^39,40^. Other insecticides, such as imidacloprid, chlorantraniliprole and triazophos have also lead to significant effects on the life cycle of the white-backed planthopper^23,26,41^. Although imidacloprid significantly affects the *GRR* in various cases, the generation time (*T*) was not affected in *B. tabaci* (Hemiptera: Aleyrodidae)^40^. Lashkari *et al*.^42^ observed that sublethal concentrations have an effect on the mean generation time in *B. brassicae* (Hemiptera: Aphididae) when treated with imidacloprid. Shorabi *et al*.^32^ also pointed out that biological character of *B. tabaci* have no significant effect at low concentration of imidacloprid and buprofezin. These reports revealed that insecticide concentrations effects life history and physiological state of the species^21,35–37,43,44^.

The analysis of the plotted curves of the age-specific survival rate (*lx*) showed more decline at LC_30_ compared to LC_10_ and the control, indicating that the LC_30_ of buprofezin is more effective. The age-stage reproductive value (v_*xj*_) indicated that buprofezin at sublethal concentrations may significantly affect the duration of immature stage but have no significant effect on adult nymphs. This result could be interrelated to an invisible effect due to inhibition when treated with a sublethal concentration of buprofezin. Due to different physical and chemical processes, the pre-adult period, APOP, total pre-oviposition period (TPOP) and mean generation time (*T*) are longer when treated with buprofezin. Similar results have also been presented in various other reports^32,34,37,38^. Qu *et al*. found that sublethal concentrations of imidacloprid (0.20 mg a.i. L^−1^) can decrease the population growth of treated *Aphis glycine* (Hemiptera: Aphididae) compared to the control through reductions in the reproductive and survival rates^36,45^. In this study, we considered that hormesis is not the only important factor in terms of effects of buprofezin on *S. furcifera*. But all the biological processes may operate simultaneously after the exposure of arthropods to pesticides due to which they may develop, ultimately, hormesis and/or resistance responses against such chemicals^33,34,46^. Therefore, further research using numerous lethal and sublethal concentrations is needed to provide a detail evaluation report of nymphal responses to buprofezin in *S. furcifera*. In summary, this study found that sublethal concentrations of buprofezin affect the longevity and egg laying of parent (F0) individuals of *S. furcifera* and also cause some variation in the biological traits of the F1 generation of *S. furcifera*. However, non-stimulatory effects on copulation and hatchability were found in the F0 generation.

Overall, these observations of the present study under laboratory conditions emphasized the significance of assessing sublethal effects of the buprofezin on WBPH and to also determine how these effects may be interpreted to population dynamics in the field. Our research suggested the need to investigate further possible effects of buprofezin on WBPH in the aim to improve optimized IPM packages including this insecticide.

## Materials and Methods

### Insects and Insecticide

The WBPH (*S. furcifera*) population was initially sampled from rice fields in Xiaogan District Hubei Province, in 2014 and has been maintained on rice seedlings for 3 years at a temperature of 27 ± 1 °C, relative humidity (RH) of 70–80% and light/dark cycle of 16:8 h in a growth chamber in a laboratory at Huazhong Agricultural University without exposure to any insecticide. All experiments were performed in the above-mentioned growth chamber. Buprofezin (97.4%, technical grade) was purchased from Jiangsu Anpon Electrochemical Co., Ltd. China.

### Bioassay

A bioassay test was carried out using the rice seedling dipping method with slight modifications^46,47^. Briefly, a stock solution of buprofezin (97.4%) was prepared in acetone and then serially diluted with water containing 0.1% Triton X-100 for five dilutions (16, 8, 4, 2, and 1 mg a.i. L^−1^). Rice plants were collected at the seedling stage and washed thoroughly with water, and then air dried at room temperature to eliminate excess water. Fifteen rice seedlings were grouped together and dipped into the serially diluted buprofezin solution treatments for 30 seconds^25^. After the treated rice stems were air dried at room temperature, the rice roots were wrapped with moistened cotton. Then, these wrapped stems were placed in 500 ml plastic cup. Forty-five 3^rd^ instar nymphs were introduced into the plastic cups using a vacuum device. Distilled water containing 0.1% Triton-X was used as a control. Both control and treatments were replicated three times for each serial dilution. The treated and control insects were kept in a plant growth chamber maintained at a temperature of 27 ± 1 °C, RH of 70 ± 1% and a light/dark cycle (L:D) of 16:8 h. Mortality was recorded after 120 h. Individual nymphs were considered dead if they did not show movement after being slightly pushed with a soft brush.

### Evaluation of sublethal effects of buprofezin on life history traits of F0 *S. furcifera*

The sublethal effects of buprofezin on the life cycle parameters of *S. furcifera* were followed by Liu and Han method with slight modifications^47^. Approximately 800–1000 adult WBPH were transferred to a clean cage with fresh and healthy rice to lay their eggs upon. After 24 h, the rice seedlings were removed and placed in another cage; these rice seedlings were retained for a number of days for nymphs to hatch out of the eggs and until these nymphs had developed into the 3^rd^ instar. These 3^rd^ instar nymphs were used as F0 generation individuals. Approximately 200–300 3^rd^ instars were transferred to and reared separately in a glass tube containing rice seedlings dipped in a sublethal concentration (LC_10_ and LC_30_) of buprofezin. The live pests were collected after 5 days. The control pests were fed rice seedlings not treated insecticide. Each surviving pest was then transferred to a separate clean tube with a healthy rice stem, and each glass tube was numbered. As the nymphs became adult males and females, they were paired (40–60 per treatment) at once in a glass tube containing a single fresh rice seedling and kept under controlled temperature (27 ± 1 °C), RH (70 ± 1%) and light/dark cycle (16:8 h). The rice seedlings in each tube were changed every day during experiment. The fecundity and longevity of the couple was recorded, and measurements continued until the death of the couple. The experiment was repeated three times.

### Effects of buprofezin on life cycle traits of F1 generation individuals of *S. furcifera*

Approximately 200 1^st^ instar nymphs were selected randomly from each F0 couple as the founders of an experimental population (F1) and kept in separate tubes under the control conditions as discussed previously. These offspring were fed rice stems, and the stage and condition of the pests were observed daily. This method was followed for both the buprofezin treated and control groups. When these nymphs become adults, they were paired as described above. The population characteristics, including developmental time, longevity, fecundity and hatchability were checked daily until the couple died. The newly born nymphs were counted and discarded. Then, the rice stems were thoroughly checked using a microscope, and the number of unhatched eggs was recorded.

During this study, the following observations were noted; the developmental rate of each instar, the emergence of adults, the duration of the adult stage, mating, fecundity and hatchability. This whole experiment was repeated 3 times.

### Statistical analysis

The Probit-MSChart^48^ program was used for probit analysis of the concentration-response data. The raw data of the life table of each *S. furcifera* individual was analysed using the age-stage, two-sex life table procedure^28,29^. The basic life-table parameters, such as age-stage survival rate (*s*_*xj*_), age-specific survival rate (*l*_*x*_), reproductive value (*v*_*xj*_), intrinsic rate of increase (*r*), finite rate of increase (*λ*), net reproductive rate (*R*_0_) and mean generation time (*T*), were analysed using the computer program TWOSEX-MS Chart^49^. The variances and standard errors of the population growth parameters were calculated using the bootstrap technique included in TWOSEX-MS Chart with 100,000 random resampling. Developmental growth, adult longevity, total preoviposition period (TPOP), adult pre-oviposition period (APOP), fecundity and population parameters (*r*, *λ*, *R*_0_, and *T*) were compared using the paired bootstrap test based on the confidence interval of the differences. Therefore, the finite rate of increase (*λ*) and intrinsic rate of increase (*r*) are the most crucial parameters for determining the potential of population growth, and these have been used to represent the fitness of populations^50–52^. Survival rate and reproductive value curves were plotted using SigmaPlot 12.0 (Systat Software Inc., San Jose, CA).
